# Trait impulsivity is associated with an increased risk of type 2 diabetes incidence in adults over 8 years of follow-up: results from the NutriNet-Santé cohort

**DOI:** 10.1186/s12916-024-03540-7

**Published:** 2024-08-15

**Authors:** Carlos Gómez-Martínez, Pauline Paolassini-Guesnier, Léopold Fezeu, Bernard Srour, Serge Hercberg, Mathilde Touvier, Nancy Babio, Jordi Salas-Salvadó, Sandrine Péneau

**Affiliations:** 1 Universitat Rovira i Virgili, Departament de Bioquímica i Biotecnologia, Alimentació, Nutrició, Desenvolupament i Salut Mental (ANUT-DSM), Reus, Spain; 2 Institut d’Investigació Sanitària Pere Virgili (IISPV), Reus, Spain; 3 Centro de Investigación Biomédica en Red de Fisiopatología de La Obesidad y Nutrición (CIBEROBN), Instituto de Salud Carlos III (ISCIII), Madrid, Spain; 4 Center of Research in Epidemiology and StatisticS (CRESS), Nutritional Epidemiology Research Team (EREN), Université Sorbonne Paris Nord and Université Paris Cité, INSERM, INRAE, CNAM, 93017 Bobigny, France

**Keywords:** Impulsivity, Prospective cohort study, Motor, Attention, Planning, Personality, Psychological traits, Type 2 diabetes

## Abstract

**Background:**

Type 2 diabetes is one of the most prevalent and preventable diseases worldwide and impulsivity, a psychological trait characterized by making quick decisions without forethought, has been suggested as a key feature for health-related conditions. However, there have been no studies examining the relationships between impulsivity and the incidence of type 2 diabetes and our aim was to assess the prospective association between trait impulsivity and the risk of developing type 2 diabetes.

**Methods:**

A prospective observational study design was conducted between May 2014 and February 2023 within the NutriNet-Santé cohort. A web-based platform was used to collect data from the French adult population, with voluntary enrollment and participation. Of the 157,591 adults (≥ 18 years old) participating in the NutriNet-Santé study when impulsivity was assessed, 109,214 participants were excluded due to prevalent type 1 or 2 diabetes or missing data for impulsivity or follow-up data for type 2 diabetes. Trait impulsivity, and the attention, motor, and non-planning subfactors, were assessed at baseline using the Barratt Impulsiveness Scale 11. Incident type 2 diabetes was ascertained through follow-up. Medical information was reviewed by NutriNet-Santé physician experts to ascertain incident diabetes cases based on the ICD-10. Cox regression models, using hazard ratios and 95% confidence intervals (HR [95% CI]), were performed to evaluate associations between impulsivity per 1 standard deviation increment and type 2 diabetes risk, adjusting by recognized confounders.

**Results:**

Of the 48,377 individuals studied (women 77.6%; age at baseline = 50.6 year ± 14.5 years), 556 individuals developed type 2 diabetes over a median follow-up of 7.78 (IQR: 3.97–8.49) years. Baseline impulsivity was associated with an increased risk of type 2 diabetes incidence (HR = 1.10 [1.02, 1.20]). The motor impulsivity subfactor was positively associated with type 2 diabetes risk (HR = 1.14 [1.04, 1.24]), whereas no associations were found for attention and non-planning impulsivity subfactors.

**Conclusions:**

Trait impulsivity was associated with an increased type 2 diabetes risk, mainly driven by the motor impulsivity subfactor. If these results are replicated in other populations and settings, trait impulsivity may become an important psychological risk factor to be considered in the prevention of type 2 diabetes.

**Cohort registration:**

Name of registry: The NutriNet-Santé Study. A Web-based Prospective Cohort Study of the Relationship Between Nutrition and Health and of Dietary Patterns and Nutritional Status Predictors.

Cohort registration number: NCT03335644.

Date of registration: October 11, 2017.

URL: https://clinicaltrials.gov/ct2/show/NCT03335644

**Supplementary Information:**

The online version contains supplementary material available at 10.1186/s12916-024-03540-7.

## Background

Type 2 diabetes is a chronic metabolic disease characterized by insulin resistance and elevated levels of blood glucose, with a worldwide prevalence of 422 million people and around 1.6 million deaths attributable to this medical condition yearly [1].

Genetic predisposition and different lifestyle behaviors have been recognized as risk factors for diabetes [2]. Personality traits predispose individuals to act within a range of behaviors [3] and have also been suggested as risk factors for different health outcomes, including diabetes [4]. Personality traits are relatively stable characteristics across the lifespan but can also be modified through psychological-based interventions [5, 6].

Trait impulsivity, in particular, is a personality trait characterized by difficulties with sustained attention, rapid motor reactions, and lack of planning [7], and has been linked to impaired inhibitory processes and higher reward sensitivity [8]. Some studies have found associations between trait impulsivity, poor diet quality [9], higher body mass index (BMI) [10], and cardiometabolic risk [11]. Therefore, impulsivity could be expected to increase the risk of type 2 diabetes incidence. However, to the best of our knowledge, there has not been any prospective exploration of associations between impulsivity and the risk of developing type 2 diabetes. A few cross-sectional studies have been performed and produced promising results [12, 13]. In one study, higher glucose levels were associated with lower inhibitory control (i.e., higher behavioral impulsivity), especially in participants with prediabetes [12]. Participants with prediabetes also exhibited a greater impulsive reward sensitivity compared to healthy control individuals [13]. Similar associations were observed in individuals with type 2 diabetes, where higher HOMA-insulin resistance and glycated hemoglobin levels were associated with poorer performance on behavioral cognitive control and decision-making, respectively [14, 15]. Finally, both higher trait and behavioral impulsivity were found to be associated with poor diabetes management [16, 17].

As impulsivity has been suggested to play a role in diabetes status and control, we hypothesize that those individuals with high trait impulsivity will have an increased risk of developing type 2 diabetes. Therefore, the main aim of this work was to assess the associations between baseline trait impulsivity and the risk of type 2 diabetes incidence over 8 years of follow-up in the NutriNet-Santé cohort.

## Methods

### Study design and population

A prospective study design was performed in the context of the NutriNet-Santé study cohort, a web-based observational study with the objective to study relationships between nutrition and health, as well as the determinants of eating behavior and health status. The NutriNet-Santé recruitment started in May 2009 and currently has an open ongoing enrolment. Volunteers are recruited via multimedia campaigns from the general French population and are included if they are ≥ 18 years old, speak French fluently, and have internet access. They are followed using a personal account on the study website (https://etude-nutrinet-sante.fr/), through which they provide detailed information by answering multiple questionnaires.

At inclusion, participants complete several self-report web-based questionnaires to assess their diet, physical activity, anthropometric measures, lifestyle characteristics, socioeconomic conditions, and health status. Participants then complete this same set of questionnaires every year after inclusion. Another set of optional questionnaires related to determinants of eating behaviors, nutritional status, and specific health-related aspects is sent to every participant each month. More information on the study protocol can be found on the following website: https://info.etude-nutrinet-sante.fr/siteinfo/, where the detailed study rationale, design, and methods are provided [18]. The study protocol was registered at https://www.clinicaltrials.gov/ (NCT03335644).

All participants report an electronic informed consent. Procedures were approved by the Institutional Research Board of the French Institute for Health and Medical Research (IRB INSERM no: 0000388FWA00005831) and the Commission Nationale de l’Informatique et des Libertés (CNIL no: 908450 and 909,216). The study accomplishes the Declaration of Helsinki standards.

### Impulsivity

Trait impulsivity was assessed using the validated French version of the Barratt Impulsiveness Scale (BIS-11) [19], derived from the BIS-10 [20]. The BIS-11 is the most used questionnaire to assess trait impulsivity in both research and clinical practice [7]. This questionnaire was administered between May and November 2014. The BIS-11 is a 30-item self-reported questionnaire with a 4-point Likert scale scoring, ranging from “rarely/never” (1 point) to “almost always/always” (4 points). The total BIS-11 score, as well as their impulsivity subfactors (attentional, motor, and non-planning), were obtained by adding their respective items. The range of impulsivity scores was as follows: BIS-11 total score (range 30–120), attentional subfactor (range 8–32), motor subfactor (range 11–44), and non-planning subfactor (range 11–44). Higher values in the scores reflect higher impulsivity. The *α* Cronbach value for the total score was 0.77, indicating an acceptable internal consistency.

### Type 2 diabetes

Type 2 diabetes status was assessed using a multisource approach (Additional file 1: Supplementary Method 1). Incident cases of type 2 diabetes and medication for this disease were recorded through yearly health questionnaires, a specific health check-up questionnaire every 6 months, or at any time spontaneously through the NutriNet-Santé platform. Medical information was reviewed by NutriNet-Santé physician experts to ascertain incident diabetes cases. Furthermore, this medical record data was linked to the Système National d’Information Inter-Régimes de l’Assurance Maladie (SNIIRAM) from the Caisse Nationale de l’Assurance Maladie of the French national insurance system where participants medication and medical consultation history was available. The SNIIRAM uses the International Chronic Diseases Classification Clinical Modification 10th Revision (ICD-10) [21] to ascertain type 2 diabetes incident cases. The first incident type 2 diabetes case was considered between the impulsivity assessment and 8th February 2023.

### Covariates

Potential confounders of the relation between trait impulsivity and type 2 diabetes were collected. We used the data closest to the date of completion of the BIS-11. Selected confounders were sociodemographics: sex (male, female), age (years), and educational level (less than high school degree, < 2 years after high school degree, ≥ 2 years after high school degree); lifestyle: smoking status (never, former, current), physical activity (low, medium, high) using the International Physical Activity Questionnaire (IPAQ) [22], energy intake without alcohol (kcal/day), alcohol intake (g/day) using 24 h-dietary records, and diet quality using the simplified Programme National Nutrition Santé—Guidelines Score 2 (sPNNS-GS2) [23]; personal history of disease: prevalence or medication use for hypertension (no, yes), hypercholesterolemia (no, yes), and hypertriglyceridemia (no, yes); family history of diabetes disease (no, yes); depressive symptomatology (no, yes) using the self-reported Center for Epidemiologic Studies Depression Scale questionnaire (CES-D) [24]; and anthropometrics: BMI (kg/m^2^).

### Statistical analysis

For this study, we included participants from the NutriNet-Santé cohort who completed the BIS-11 and did not have prevalent type 1 or 2 diabetes diagnosed at baseline. Covariates with missing values were handled using Multiple Imputation by Chained Equations (MICE) by fully condition specification (Additional file 1: Supplementary Method 2).

Differences in trait impulsivity scores between the excluded participants with prevalent type 2 diabetes and those included in the final population were assessed using a *t*-test. A comparison of baseline population characteristics between included and excluded participants was performed, using *t*-test or chi-square, as appropriate. Baseline participant characteristics are presented as numbers and percentages for qualitative variables and as mean ± standard deviation (SD) for quantitative variables. Comparisons across trait impulsivity categories (low, medium, and high) were based on chi-square for categorical variables and one-way ANOVA for quantitative variables.

Cox regression models, using hazard ratios and 95% confidence interval (HR 95% CI), were performed to evaluate the linear associations between 1 SD increment of trait impulsivity (and attention, motor, and non-planning subfactors) and the incidence of type 2 diabetes, over 8 years of follow-up. Participants contributed person-time from their impulsivity assessment until the date of type 2 diabetes event, date of last follow-up, date of death, or 8th February 2023, whichever occurred first, and incidence rates were estimated. A parsimonious model was run and adjusted at baseline for age (as timescale) and sex. The main model was further adjusted at baseline for educational level (with a logarithmic time interaction), smoking status, physical activity, energy intake without alcohol, alcohol intake, and diet quality. The rationale for covariates selection is described in Additional file 2: Table S1 [9, 23, 25–40] and Additional file 2: Table S2 [10, 24, 41–46].

Linearity assumptions between total impulsivity, and its subfactors, with type 2 diabetes incidence were verified using restricted cubic spline functions. If the associations were non-linear (motor subfactor only), a correction was applied using a logarithmic base 10 transformation. Impulsivity categories were used to estimate cumulative hazard risks (Additional file 3: Fig. S1). Impulsivity categories were determined by using specified cut-offs [7]: low (< 52), medium (≥ 52 and ≤ 71), and high (> 71). An exploratory analysis was conducted studying the associations between trait impulsivity categories and the incidence of type 2 diabetes, using Cox regression models. Pearson correlation coefficients were assessed to confirm the absence of collinearity between the continuous variables included in the main model (Additional file 2: Table S3). Schoenfeld residuals were computed to confirm the risk of proportionality hazard assumptions. Covariates with non-proportional hazard risk were corrected in the main analyses by establishing an interaction by logarithmic time (education level only). Furthermore, Schoenfeld residuals were tested again correcting for non-proportional hazard covariates.

Sensitivity analyses were performed to verify the robustness of the findings. Associations between impulsivity and type 2 diabetes risk were analyzed using stratified estimates for non-proportional hazard covariates and without a correction for these non-proportional covariates. A total of six additional models were evaluated with the aim of studying various confounding factors. The exclusion of incident cases of type 2 diabetes during the first 2 years of follow-up was also assessed. Interactions were tested using the likelihood ratio test for sex, age (< 60 years, ≥ 60 years), overweight (BMI: < 25, ≥ 25 kg/m^2^), and diet quality (sPNNS-GS2: median to determine low and high diet quality, by sex). A post hoc mediation analysis was conducted to assess the role of baseline BMI in the association between a 1 SD increase in total trait impulsivity and the risk of developing type 2 diabetes. Linear regressions were used to determine the association between impulsivity (exposure) and BMI (mediator), and Cox regressions were used to determine the association of impulsivity and BMI with incident type 2 diabetes (outcome). BMI was fixed as its mean. The model was adjusted for the same covariates as the main model, and confounders were fixed either at the mode (sex, education, smoking status, and physical activity) or at the mean (energy intake, alcohol intake, and diet quality). Analyses were performed using the bootstrapping decomposition method (seed: 4500; replicates: 1000).

Analyses were performed with STATA 14. The STATA med4way package [47] was used for mediation analyses. Statistically significant results were considered when *P* value was < 0.05.

## Results

### Description of the study population

**Fig. 1:**
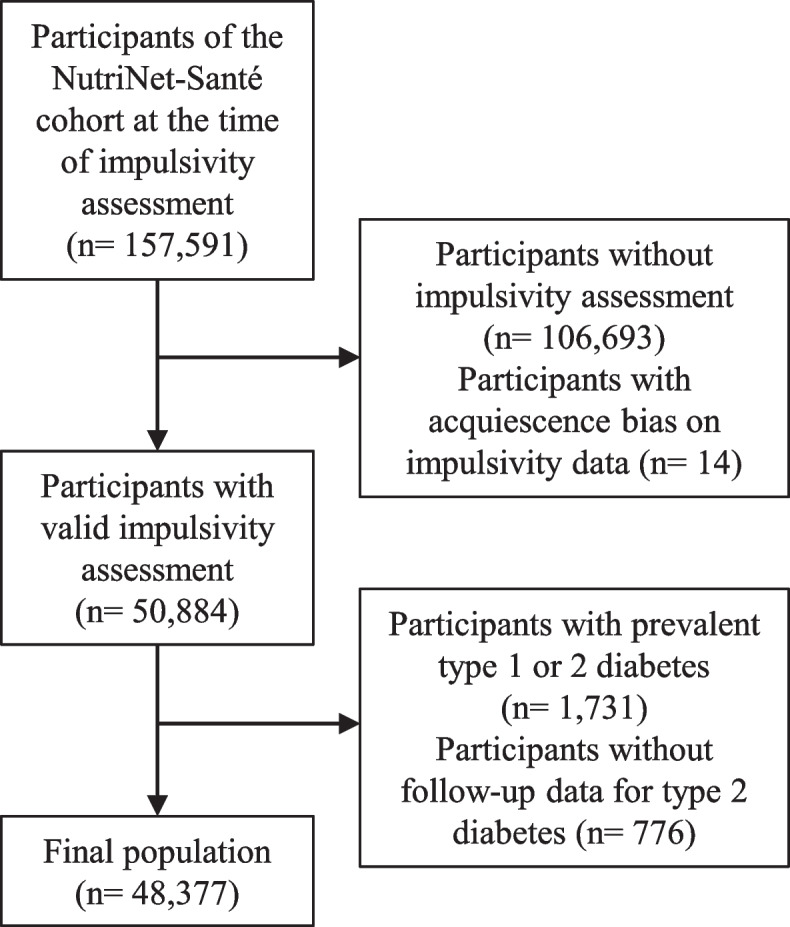
Flowchart of the studied population

Among the 157,591 individuals included in the NutriNet-Santé study at the time of impulsivity assessment, a total of 48,377 participants were included in the present analyses and of these, 556 developed type 2 diabetes (Fig. 1).

Comparison of baseline population characteristics between included and excluded participants can be found in Additional file 2: Table S4. Baseline characteristics of the studied population were shown in Table 1. Mean age was 50.4 (SD: 14.6) years, and around three-quarters of the population were women. Compared with individuals presenting lower impulsivity, those with higher impulsivity were more likely to be younger, female, and former or current smokers; to have a lower level of education, physical activity, and diet quality; and to have a higher alcohol consumption, BMI, and prevalence or medication use of hypertension, hypertriglyceridemia, and depressive symptomatology. The median follow-up was approximately 8 (median: 7.78; interquartile range: 3.97–8.49) years (person-years: 297,027), and the incidence rate (95% CI) was 1.87 (1.72, 2.03) for 1000 person-years.

**Table 1:** Baseline characteristics of the study population by trait impulsivity categories, NutriNet-Santé cohort, France, 2014–2023 (*n* = 48,377)

Characteristics	All participants	Trait impulsivity			*P* value^*^
		Low (*n* = 8659)	Medium (*n* = 36,743)	High (*n* = 2975)	
Age (years)	50.63 ± 14.56^†^	51.11 ± 14.06	50.59 ± 14.60	49.78 ± 15.37	< 0.001
Sex (female)	37,568 (77.66)^‡^	6244 (72.11)	28,863 (78.55)	2461 (82.72)	< 0.001
Educational level (*n* = 48,278)					< 0.001
Less than high school degree	1032 (2.14)	120 (1.39)	779 (2.13)	133 (4.48)	
< 2 years after high school degree	14,108 (31.39)	2167 (25.09)	10,753 (29.36)	1188 (40.01)	
≥ 2 years after high school degree	33,091 (68.61)	6351 (73.52)	25,092 (68.51)	1648 (55.51)	
Smoking status (*n* = 48,376)					< 0.001
Never	22,333 (46.17)	4625 (53.41)	16,687 (45.42)	1021 (34.32)	
Former	20,899 (43.20)	3423 (39.53)	16,051 (43.69)	1425 (47.90)	
Current	5144 (10.63)	611 (7.06)	4004 (10.90)	529 (17.78)	
Physical activity (IPAQ) (*n* = 48,314)					< 0.001
Low	11,025 (22.83)	8408 (22.92)	84,088 (22.92)	751 (25.37)	
Medium	20,248 (41.94)	3561 (41.20)	15,501 (42.26)	1186 (40.07)	
High	17,009 (35.23)	3217 (37.22)	12,769 (34.81)	1023 (34.56)	
Energy intake without alcohol (kcal/day) (*n* = 45,080)	1787.29 ± 474.28	1789.09 ± 474.73	1786.23 ± 471.91	1795.35 ± 502.41	0.59
Alcohol intake (g/day) (*n* = 45,080)	7.92 ± 12.01	7.21 ± 11.12	8.05 ± 12.10	8.66 ± 13.22	< 0.001
Diet quality (sPNNS-GS2; range: − 17 to 13.5) (*n* = 44,318)	1.28 ± 3.56	1.41 ± 3.55	1.27 ± 3.54	0.95 ± 3.80	< 0.001
Body mass index (kg/m^2^) (*n* = 48,212)	23.86 ± 4.34	23.65 ± 4.16	23.87 ± 4.36	24.41 ± 5.00	< 0.001
Hypertension prevalence and/or medication	6400 (13.23)	1131 (13.06)	4818 (13.11)	451 (15.16)	0.006
Hypercholesterolemia prevalence and/or medication	8719 (18.02)	1545 (17.84)	6618 (18.01)	556 (18.69)	0.58
Hypertriglyceridemia prevalence and/or medication	1657 (3.43)	285 (3.29)	1243 (3.38)	129 (4.34)	0.017
Family history of diabetes (*n* = 47,996)	9689 (20.19)	1717 (19.97)	7370 (20.21)	602 (20.53)	0.78
Depressive symptomatology (*n* = 19,464)	2359 (12.12)	263 (7.71)	14,887 (12.14)	288 (24.68)	< 0.001

Abbreviations: *IPAQ*, International Physical Activity Questionnaire; *sPNNS-GS2*, simplified Programme National Nutrition Santé—Guidelines Score 2

Trait impulsivity categories were determined using the following cut-offs: low (< 52), medium (≥ 52 and ≤ 71), and high (> 71), based on the Barratt Impulsiveness Scale 11 questionnaire

^*^P value showing comparisons between categories of trait impulsivity (low, medium, high) based on chi-square for categorical variables and ANOVA for quantitative variables

^†^Mean ± SD (all such values)

^‡^*n* (%) (all such values)

Participants excluded due to prevalent type 2 diabetes at baseline exhibited higher trait impulsivity scores (mean: 59.83; SD: 8.48) compared to the final population (mean: 58.70; SD: 7.98) analyzed (*P* < 0.001). No statistically significant difference was observed in the total impulsivity score between participants with prevalent (mean: 59.83; SD: 8.48) and incident (mean: 59.35; SD: 8.06) type 2 diabetes (*P* = 0.15).

### Associations between trait impulsivity and type 2 diabetes

Restricted cubic splines suggested a linear relationship between total impulsivity and type 2 incident diabetes (*P* = 0.51) (Additional file 3: Fig. S2).

**Fig. 2:**
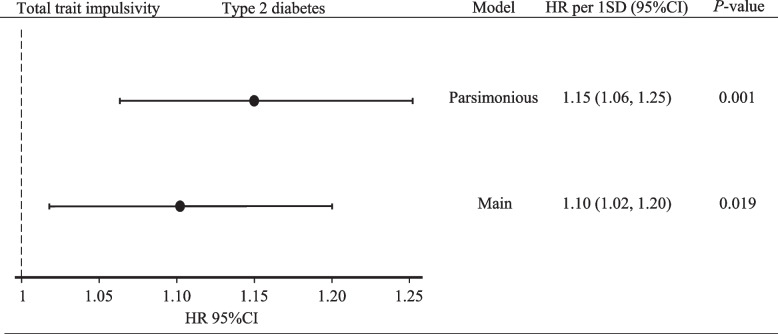
Associations between trait impulsivity and type 2 diabetes risk, NutriNet-Santé cohort, France, 2014–2023 (*n* = 48,377). Abbreviations: HR per 1 SD (95% CI), hazard ratio per 1 standard deviation increment and 95% confidence interval. Cox regression analyses were performed using hazard ratios and 95% CI to assess associations between 1 SD increment of total trait impulsivity and the risk of type 2 diabetes incidence over a median follow-up of 8 years in the NutriNet-Santé cohort. Total population (*n* = 48,377) and type 2 diabetes incident cases (*n* = 556). Person-years = 297,027 and incidence rate = 1.87 (95% CI: 1.72, 2.03) per 1000 person-years. Parsimonious model: adjusted for baseline sex and age (time scale). Main model: parsimonious model + baseline education level (less than high school degree, < 2 years after high school degree, ≥ 2 years after high school degree), smoking status (never, former, current smoker), physical activity (International Physical Activity Questionnaire: high, moderate, low), energy intake without alcohol (kcal/day), alcohol intake (g/day), and diet quality (simplified Programme National Nutrition Santé—Guidelines Score 2). Non-proportional hazard risk covariates were corrected by adding a logarithmic time interaction (educational level only)

In both the parsimonious and main model, a positive linear significant association was found between a 1 SD increment of impulsivity and incidence of type 2 diabetes (main model; HR [95% CI] = 1.10 [1.02, 1.20]; *P* = 0.019) (Fig. 2). The associations between trait impulsivity categories and the incidence of type 2 diabetes were not significant (Additional file 2: Table S5).

**Fig. 3:**
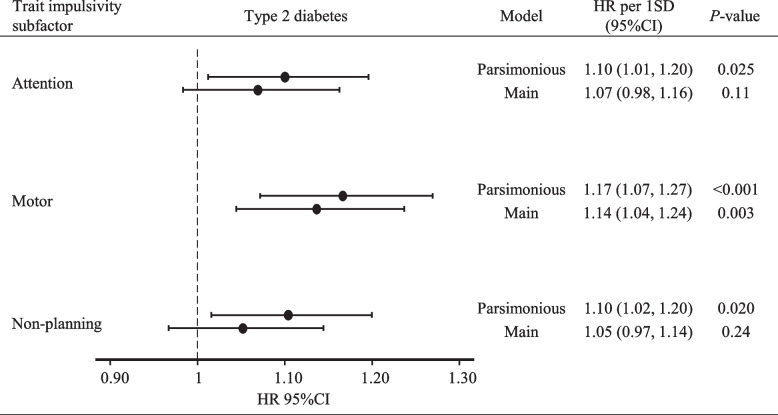
Associations between trait impulsivity subfactors and type 2 diabetes risk, NutriNet-Santé cohort, France, 2014–2023 (*n* = 48,377). Abbreviations: HR per 1 SD (95% CI), hazard ratio per 1 standard deviation increment and 95% confidence interval. Cox regression analyses were performed using hazard ratios and 95% CI to assess associations between 1 SD increment of attention, motor, and non-planning trait impulsivity subfactors and the risk of type 2 diabetes incidence over a median follow-up of 8 years in the NutriNet-Santé cohort. The attention impulsivity subfactor was log10 transformed due to potential non-linear association (*P* = 0.03). Total population (*n* = 48,377) and type 2 diabetes incident cases (*n* = 556). Person-years = 297,027 and incidence rate = 1.87 (95% CI: 1.72, 2.03) per 1000 person-years. Parsimonious model: adjusted for baseline sex and age (time scale). Main model: parsimonious model + baseline education level (less than high school degree, < 2 years after high school degree, ≥ 2 years after high school degree), smoking status (never, former, current smoker), physical activity (International Physical Activity Questionnaire: high, moderate, low), energy intake without alcohol (kcal/day), alcohol intake (g/day), and diet quality (simplified Programme National Nutrition Santé—Guidelines Score 2). Non-proportional hazard risk covariates were corrected by adding a logarithmic time interaction (educational level only)

In the parsimonious model, all impulsivity subfactors (attention, motor, and non-planning) were associated with an increased risk of type 2 diabetes incidence, although only motor impulsivity remained significantly related in the main model (HR [95% CI] = 1.14 [1.04, 1.24]; *P* = 0.003) (Fig. 3).

Given the non-proportional hazard estimates observed for educational level (Additional file 2: Table S6; Additional file 2: Table S7), sensitivity analyses were performed with a stratification on educational level and without correction for this covariate. In both analyses, the directionality and significance of the associations between total impulsivity, its subfactors, and type 2 diabetes were maintained (Additional file 2: Table S8; Additional file 2: Table S9). The associations between total trait impulsivity and type 2 diabetes were also maintained after the adjustment of sociodemographic and lifestyle confounders (Additional file 2: Table S10). However, the additional adjustment for personal and familiar history of disease, as well as for depressive symptomatology, attenuated the relationships showing borderline non-significant results (all *P* ≤ 0.075). More specifically, after the inclusion of BMI in the models, the association was largely attenuated becoming non-significant (*P* = 0.53). When excluding participants with early incident type 2 diabetes (first 2 years of follow-up), the associations were also attenuated (*P* = 0.11) (Additional file 2: Table S11). Assessed interactions for sex, age, overweight, and diet quality showed no significant results (all *P* > 0.20). The results of the mediation analysis by BMI in the association between trait impulsivity and the risk of developing type 2 diabetes showed a significant total effect (HR [95% CI] = 1.10 [1.00, 1.19]; *P* = 0.048), a borderline significant controlled direct effect when BMI was fixed at its mean (HR = 1.09 [0.99, 1.18]; *P* = 0.070), and a significant pure indirect effect (HR [95% CI] = 1.02 [1.01, 1.03]; *P* < 0.001) indicating a strong mediation effect of BMI (Additional file 3: Fig. S3).

## Discussion

To the best of our knowledge, this is the first study investigating long-term relationships between impulsivity and type 2 diabetes incidence. In the present study, conducted in the context of a large population, baseline trait impulsivity was significantly associated with an increased risk of type 2 diabetes, independently of several recognized confounding factors.

Psychological aspects have been highlighted as major characteristics for the establishment of diseases [4]. Psychological traits predispose human behavior based on established patterns of emotions and cognitive processes that interact with genetic, biological, cultural, and social spheres [3]. Therefore, it seems reasonable that psychological traits, such as an impulsive proneness which has been associated with unhealthy behaviors and glycemic dysregulations [11, 16, 17], may play a role in the development of glucose-related diseases such as type 2 diabetes.

Unfortunately, there is a lack of studies analyzing the associations between personality traits and the incidence of type 2 diabetes, and none of the studies has examined these associations evaluating trait impulsivity. Remarkably, the present work shows an association between trait impulsivity and the risk of developing type 2 diabetes. Our data support previous cross-sectional studies in populations with or at risk of developing type 2 diabetes where an inverse relationship between behavioral impulsivity levels and glycemic status [12–15], and poor diabetes control [16, 17] have been reported.

The present results are also in line with those of a pooled project including 5 prospective cohorts and 34,914 adults from the USA and the UK where higher trait conscientiousness was linearly associated with lower type 2 diabetes incidence and its caused-related mortality [48]. Trait impulsivity has been proposed as an antagonist of conscientiousness, which is described as the propensity to be self-controlled, responsible, and well-organized [49]. The associations shown in the present work between impulsivity and type 2 diabetes could be explained by the idiosyncratic neurological impulsivity network, which may lead to impulsive behaviors guided by the trait impulsivity proneness. The characteristic impulsive neural pathway involves the prefrontal cortex (high-order cognitive system), ventral striatum and nucleus accumbens (reward-dopaminergic system), and amygdala (emotional-limbic system) regions [50]. The interaction between this network and impulsivity traits drives individuals to have a great urgency to respond to their positive and negative emotional states and a high sensitivity to immediate rewards, combined with a lack of prefrontal cortex capacity to restrain the need for immediate gratification or to avoid negative emotions [51, 52]. Consequently, this interplay between trait and neurological impulsivity predisposes individuals to externalize impulsive behaviors characterized by acting without considering the consequences of their behavior [51, 52]. These mechanisms may also explain the positive associations found between impulsivity measures and major risk factors for type 2 diabetes such as diet quality [9], BMI [10], insulin resistance [14], and hyperglycemia [12].

Some lifestyle behaviors, such as poor diet quality, have been shown to increase cytokines production in adipose tissue and liver, promoting insulin resistance [53]. Results from the Nurses’ Health Studies, which followed more than 200,000 participants, indicated that the consumption of unhealthy food was associated with an increased incidence of type 2 diabetes [54]. Trait impulsivity has also been shown to be inversely associated with the adherence to healthy dietary patterns [9]. In our analyses, the adjustment for different food-related variables did not change the directionality and significance of the associations between impulsivity and type 2 diabetes incidence, but we cannot completely discard some residual confounding effects. When BMI was included as a covariate in the models, the association was largely attenuated and became non-significant. Mediation analyses confirmed a substantial mediating effect of BMI on the association between trait impulsivity and type 2 diabetes incidence. While the indirect effect through BMI was significant, the direct effect did not reach statistical significance, shedding light on the observed attenuation. Impulsivity has previously been associated with higher odds of obesity in the NutriNet-Santé study and with increasing BMI levels as shown by a meta-analysis [10, 55]. Adiposity has a recognized central role in the development of type 2 diabetes as higher BMI has been shown to induce low-grade inflammation and decrease insulin sensitivity, increasing the risk of hyperglycemia [56]. In addition, longitudinal studies show that negative psychological factors generally precede and predict faster rate of weight gain rather than the opposite [57], suggesting a unidirectional association between impulsivity and BMI on the risk of type 2 diabetes incidence.

When considering subtraits of impulsivity, we observed that motor personality was the only subfactor consistently associated with type 2 diabetes incidence in the main models, whereas attention and non-planning impulsivity subfactors did not show significant associations. Impulsivity subtraits are attributable to different cognitive control subsystems [58]. In particular, the motor impulsivity subfactor, which involves acting without thinking [7], has been found to be more strongly associated with the activation of neural networks related to cognitive control compared with the attention and non-planning subfactors [58]. These neural networks have been proposed to be key neural bases for behavioral impulsivity [8]. Therefore, and in consideration of the fact that personality traits are precursors of behaviors [3], the results of the present study, which demonstrated a consistent association between motor trait impulsivity and the incidence of type 2 diabetes, could suggest that this subfactor may be the precursor of the deleterious relationships found between behavioral impulsivity and glucose-related measurements [12–15]. When participants with incident cases of type 2 diabetes within the first 2 years of follow-up were removed, only the motor subfactor remained associated with type 2 diabetes in the main model, suggesting a strong association for this personality subfactor. It is important to consider that this sensitivity analysis excluded around 7000 participants and almost 50% of incident cases, which considerably reduced the statistical power. Given that half of the cases occurred during the first 2 years of follow-up, it is possible that reverse causation may have occurred between impulsivity and type 2 diabetes. However, the impulsivity trait is theoretically established early in life [52] which reduces the likelihood of such reverse causation.

The main strengths of the present analyses were the novelty of the assessed relationships, the large population studied, and the long period of follow-up, but some limitations deserve to be mentioned. First, the observational design did not allow to establish causal relationships. Second, some additional residual confounding bias could exist, although models were adjusted by several and recognized confounders and multiple sensitivity analyses were performed to check the robustness of the findings. Third, trait impulsivity was a self-reported measure, but the questionnaire employed was the most widely used assessment of trait impulsivity and was validated for the French population. Addressing impulsive eating more specifically could be of great interest given that disinhibited eating behavior has been positively associated with insulin resistance [59]. Fourth, the type 2 diabetes incidence rate per 1000 person-years observed in our study (1.87) was low compared to the incidence rate (between 7.74 and 8.97 for the period 2012–2020) found in a recent study involving more than 20 million French individuals [60]. The rate observed in our study may be partly explained by the voluntary enrollment of the participants in the NutriNet-Santé study where more women and more participants with higher education, higher income, and professional status are found than in the general French population [61]. These participants may be more likely to have high health awareness and a stronger interest in nutrition which in turn is associated with a lower risk of developing metabolic diseases. The selection bias indicates that caution should be exercised when extrapolating the results to the general population. For example, higher impulsivity values are usually reported in men and our population was predominantly women. However, a sex interaction was tested and not found.

The clinical relevance of this study should be noted. Higher levels of impulsivity have been associated with an increase in the risk of highly prevalent disorders with substantial public health burden such as overeating and obesity [10], eating disorders [62], substance-related disorders [50], and psychiatric conditions [51]. The present work further extends these findings by highlighting the increased risk of developing type 2 diabetes associated with higher impulsivity. Emphasizing positive psychological characteristics could be potential strategies for the primary and secondary prevention of type 2 diabetes. In particular, mindfulness which has showed an inverse association with impulsivity could be beneficial [6, 63]. Additionally, the need for cognition (NFC) has been identified as a protective psychological factor for diabetes self-management and glycemic control [64]. Reducing the intensity of negative emotions in patients with type 2 diabetes is another potential factor of interest given its mediating effect on the relationship between trait impulsivity and executive function [65]. It is also important to acknowledge that impulsive behaviors can sometimes be strategic responses based on individual needs or motivations and therefore may serve adaptive purposes in certain contexts [66]. Therefore, further research is needed to explore the complex associations between impulsivity and chronic diseases, considering both potential benefits and risks.

## Conclusions

To conclude, trait impulsivity was associated with an increased risk of type 2 diabetes incidence over 8 years of follow-up in a large French cohort. Specifically, the personality subfactor of motor impulsivity was found to be an important feature to consider for the onset of type 2 diabetes. If these results are confirmed in other populations and settings, trait impulsivity could be a promising psychological risk factor to consider for the prevention of type 2 diabetes.

### Supplementary Information

Additional file 1: Supplementary Method 1 Incident type 2 diabetes ascertainment in NutriNet-Santé and biological data assessment. Supplementary Method 2 Multiple Imputation by Chained Equations

Additional file 2: Table S1 Rationale for selected confounders in the main model. Table S2 Rationale for selected confounders in sensitivity analyses. Table S3 Pearson correlations between continuous variables included in the main model. Table S4 Baseline characteristics of the study population comparing included and excluded participants. Table S5 Associations between categories of trait impulsivity and the risk of developing type 2 diabetes. Table S6 Assessment of proportional hazard risk assumptions between total trait impulsivity and risk of developing type 2 diabetes, with and without correction for non-proportional hazard risks covariates. Table S7 Assessment of proportional hazard risk assumptions between impulsivity subfactors and risk of developing type 2 diabetes, with and without correction for proportional hazard risks covariates. Table S8 Associations between total and subfactors of trait impulsivity and risk of developing type 2 diabetes over 8 years using stratified estimates for non-proportional hazard risk covariates. Table S9 Associations between total and subfactors of trait impulsivity and risk of developing type 2 diabetes over 8 years without proportional hazard risk correction. Table S10 All models of associations between total and subfactors of trait impulsivity and risk of developing type 2 diabetes over 8 years. Table S11 Associations between total and subfactors of trait impulsivity and risk of developing type 2 diabetes over 8 years excluding incident cases in the first 2 years of follow-up

Additional file 3: Fig. S1 Cumulative hazards between categories of total trait impulsivity and risk of developing type 2 diabetes. Fig. S2 Restricted cubic splines between trait impulsivity and risk of developing type 2 diabetes. Fig. S3 Mediation analysis of body mass indexlevels in the associations between total trait impulsivity and the risk of developing type 2 diabetes

## Data Availability

Researchers from public institutions can submit a collaboration request to Dr. Mathilde Touvier via collaboration@etude-nutrinet-sante.fr, including information on the institution and a brief description of the project. All requests will be reviewed by the steering committee of the NutriNet-Santé study. If the collaboration is accepted, a data access agreement will be necessary, and appropriate authorizations from competent administrative authorities may be needed. In accordance with existing regulations, no personal data will be accessible. The analysis code can be requested from the authors.

## Abbreviations

BIS-11: Barratt Impulsiveness Scale
BMI: Body mass index
CES-D: Center for Epidemiologic Studies Depression Scale questionnaire
ICD-10: International Chronic Diseases Classification Clinical Modification 10th Revision
IPAQ: International Physical Activity Questionnaire
MICE: Multiple Imputation by Chained Equations
NFC: Need for cognition
SD: Standard deviation
SNIIRAM: Système National d’Information Inter-Régimes de l’Assurance Maladie
sPNNS-GS2: Simplified Programme National Nutrition Santé—Guidelines Score 2
