# Supplementary material for: Trait impulsivity is associated with an increased risk of type 2 diabetes incidence in adults over 8 years of follow-up: results from the NutriNet-Santé cohort
Source: BMC Med. 2024 Aug 15;22:332. doi: 10.1186/s12916-024-03540-7 (PMC11328429; doi:10.1186/s12916-024-03540-7)
Supplement: Supplementary file 1 — Additional file 1: Supplementary Method 1 Incident type 2 diabetes ascertainment in NutriNet-Santé and biological data assessment. Supplementary Method 2 Multiple Imputation by Chained Equations [file 12916_2024_3540_MOESM1_ESM.docx]

**Supplementary Method 1.** Incident type 2 diabetes ascertainment in NutriNet-Santé and biological data assessment.

Participants were asked to declare major health events though the yearly health questionnaire, through a specific health check-up questionnaire every six months, or at any time through a specific interface on the study website. They were also asked to declare all currently taken medications and treatments via the check-up and yearly questionnaires. A search engine with embedded exhaustive Vidal drug database is used to facilitate medication data entry for the participants. Besides, our research team was the first in France to obtain the authorization by Decree in the Council of State (n°2013-175) to link data from our general population-based cohorts to medico-administrative databases of the National health insurance (SNIIRAM database). Thus, data from the NutriNet-Santé cohort are linked every year to medico-administrative databases of the SNIIRAM, providing detailed information about the reimbursement of medication and medical consultations. Participants have been informed about this linkage and had the right to revoke their given consent at any time on a dedicated interface on the study website.

Regarding T2D specifically: all 969 cases were primarily detected through the declaration by the participants of a T2D diagnosed by a physician and/or diabetes medication use, in follow-up questionnaires. The questions were: “Have you been diagnosed with T2D (if yes, indicate the date of diagnosis)” and “Are you treated for T2D?”. ATC codes considered for T2D medication were A10AB01, A10AB03, A10AB04, A10AB05, A10AB06, A10AC01, A10AC03, A10AC04, A10AD01, A10AD03, A10AD04, A10AD05, A10AE01, A10AE02, A10AE03, A10AE04, A10AE05, A10AE30, A10BA02, A10BB01, A10BB03, A10BB04, A10BB06, A10BB07, A10BB09, A10BB12, A10BD02, A10BD03, A10BD05, A10BD07, A10BD08, A10BD10, A10BD15, A10BD16, A10BF01, A10BF02, A10BG02, A10BG03, A10BH01, A10BH02, A10BH03, A10BX02, A10BX04, A10BX07, A10BX09, A10BX10, A10BX11, A10BX12.

Following a T2D diagnosis and/or medication declaration, two additional sources of information were considered for confirmation. First, the linkage with the SNIIRAM National health insurance database allowed confirming 85.7% of investigated cases (ICD-10 codes E11). The sensitivity of SNIIRAM databases is not optimal if used without self-report data: of note, about 10-15% of the French population is covered by other social security regimen and would not be correctly captured by the SNIIRAM databases. Besides, the centralization of SNIIRAM data might take up to a year, leading to delays between reported T2D information and health insurance data.

Second, as regards biological samples, a subsample of participants (n=19,800) were invited to a clinical examination, during which they provided blood and urine samples (details below). Among the participants who provided blood sample during the clinical/biological examination, 232 had elevated fasting blood glucose (i.e. >1.26 g/L). Among them, 85.3% had consistently declared a T2D diagnosis and/or medication. Elevated blood glucose alone (i.e., without any declaration of T2D diagnosis or treatment), especially if issued from a single biological measurement, was not considered specific enough to classify the participant as a T2D case.

*Biological data assessment*

During a clinical examination visit, blood samples were collected after at least a 6-h fast period and centralized and analyzed at a single laboratory (IRSA, Tours, France). Total serum cholesterol (cholesterol oxidase C8000, Abbott), high-density protein cholesterol (HDL-cholesterol) (direct accelerator C8000, Abbott), serum triglycerides (glycerol kinase C8000, Abbott), and fasting blood glucose were measured (hexokinase on C 8000 automat, Abbott, Suresnes, France). Low density protein cholesterol (LDL-cholesterol) was calculated using the Friedwald formula.

**Supplementary Method 2.** Multiple Imputation by Chained Equations.

Within the sample (n=48,377), missing values for covariates were handled using the Multiple Imputation by Chained Equations method using fully conditional specification (imputed datasets= 20; seed= 1234) for the following covariates: educational level (missing data, n= 146, 0.3%), smoking status (n= 1; <0.01%), physical activity (n= 95; 0.2%), energy intake without alcohol consumption (n= 3,297; 6.8%), alcohol intake (n= 3,297; 6.8%), diet quality (n= 4,059; 8.4%), body mass index (n= 165; 0.3%), familiar history of diabetes (n= 381; 0.8%), and depressive symptomatology (n= 28,913; 59.8%).
