# Supplementary material for: Trait impulsivity is associated with an increased risk of type 2 diabetes incidence in adults over 8 years of follow-up: results from the NutriNet-Santé cohort
Source: BMC Med. 2024 Aug 15;22:332. doi: 10.1186/s12916-024-03540-7 (PMC11328429; doi:10.1186/s12916-024-03540-7)
Supplement: Supplementary file 2 — Additional file 2: Table S1 Rationale for selected confounders in the main model. Table S2 Rationale for selected confounders in sensitivity analyses. Table S3 Pearson correlations between continuous variables included in the main model. Table S4 Baseline characteristics of the study population comparing included and excluded participants. Table S5 Associations between categories of trait impulsivity and the risk of developing type 2 diabetes. Table S6 Assessment of proportional hazard risk assumptions between total trait impulsivity and risk of developing type 2 diabetes, with and without correction for non-proportional hazard risks covariates. Table S7 Assessment of proportional hazard risk assumptions between impulsivity subfactors and risk of developing type 2 diabetes, with and without correction for proportional hazard risks covariates. Table S8 Associations between total and subfactors of trait impulsivity and risk of developing type 2 diabetes over 8 years using stratified estimates for non-proportional hazard risk covariates. Table S9 Associations between total and subfactors of trait impulsivity and risk of developing type 2 diabetes over 8 years without proportional hazard risk correction. Table S10 All models of associations between total and subfactors of trait impulsivity and risk of developing type 2 diabetes over 8 years. Table S11 Associations between total and subfactors of trait impulsivity and risk of developing type 2 diabetes over 8 years excluding incident cases in the first 2 years of follow-up [file 12916_2024_3540_MOESM2_ESM.docx]

**Table S1.** Rationale for selected confounders in the main model.

| **Covariate** | **Coding of the variable** | **Relationship with impulsivity** | **Relationship with T2D** | **Method for collection/ measurement** |
| --- | --- | --- | --- | --- |
| Age | Used as time-scale in Cox models | Impulsivity decreases with aging[1, 2] | Aging is associated with higher T2D risk[3] | Self-reported socio-demographic and lifestyle questionnaires |
| Sex | Categorical: male, female | There are different patterns of impulsivity across sex[4] | Women have greater T2D risk compared with men[5] | Self-reported socio-demographic and lifestyle questionnaires |
| Educational level | Categorical: less than high school degree, <2 years after high school degree, ≥2 years after high school degree | Higher impulsivity is associated with lower educational level[6] | Lower level of education is associated with T2D risk[7] | Self-reported socio-demographic and lifestyle questionnaires |
| Smoking status | Categorical: never smoker, former smoker, current smoker | Impulsivity is positively associated with cigarette consumption[8, 9] | Smoking status is associated with T2D risk[10] | Self-reported socio-demographic and lifestyle questionnaires |
| Physical activity | Categorical: low, moderate, high | Impulsivity is associated with patterns of physical activity[11] | Physical inactivity is associated with T2D risk[12] | Validated International Physical Activity Questionnaire[13] |
| Energy intake without alcohol | Continuous (kcal/day) without alcohol consumption | Higher impulsivity is associated with higher energy intake[14] | Total energy intake is positively associated with T2D risk[15] | 24h-dietary records linked with the NutriNet-Santé food composition database |
| Alcohol intake | Continuous (g/day) | Higher impulsivity is associated with higher alcohol intake[14] | Alcohol is positively associated with glucose metabolism in people with diabetes[16] | 24h-dietary records linked with the NutriNet-Santé food composition database |
| Diet quality | Continuous: sPNNS-GS2 score (range: −17 to 13.5) | Higher impulsivity is associated with less adherence to healthy dietary patterns and with higher adherence to unhealthy dietary patterns[14, 17] | Healthy diets are inversely associated with T2D risk[10] | 24h-dietary records linked with the NutriNet-Santé food composition database. sPNNS-GS2, simplified Programme National Nutrition Santé - Guidelines Score 2[18] |

Abbreviations: T2D, type 2 diabetes.

**Table S2.** Rationale for selected confounders in sensitivity analyses.

| **Covariate** | **Coding of the variable** | **Relationship with impulsivity** | **Relationship with T2D** | **Method for collection/ measurement** |
| --- | --- | --- | --- | --- |
| BMI | Continuous (kg/m²) | Impulsivity is associated with higher BMI[19] | BMI is an intermediary outcome between risk factors of T2D and the T2D onset[10] | Self-reported anthropometric questionnaires |
| Hypertension prevalence or medication | Categorical: yes, no | Impulsivity is associated with higher hypertension risk[20] | Hypertension is an intermediary outcome between risk factors of T2D and the T2D onset[10] | Self-reported health questionnaire |
| Hypercholesterolemia prevalence or medication | Categorical: yes, no | Impulsivity is associated with lower HDL-cholesterol levels[21] | Hypercholesterolemia is an intermediary outcome between risk factors of T2D and the T2D onset[10] | Self-reported health questionnaire |
| Hypertriglyceridemia prevalence or medication | Categorical: yes, no | Impulsivity is associated with higher triglyceride levels[21] | Hypertriglyceridemia is an intermediary outcome between risk factors of T2D and the T2D onset[10] | Self-reported health questionnaire |
| Family history of diabetes | Categorical: yes, no | Impulsivity is associated with higher risk of T2D[22].  However, to our knowledge, no evidence exists regarding family history of T2D | Family history of T2D is associated with higher risk of T2D[23] | Self-reported health questionnaire |
| Depressive symptomatology  (in sensitivity analyses due to the high number of missing data) | Categorical: yes, no | Impulsivity is positively associated with depressive symptomatology[24] | Depressive symptoms and diagnosis are associated with an increased risk of T2D[25] | Self-reported Center for Epidemiologic Studies Depression Scale questionnaire[26] |

Abbreviations: BMI, Body Mass Index; T2D, type 2 diabetes.

**Table S3.** Pearson correlations between continuous variables included in the main model.

| Characteristics | Age | Energy intake without alcohol | Alcohol intake | Diet quality |
| --- | --- | --- | --- | --- |
| Age | - |  |  |  |
| Energy intake without alcohol | -0.03*^†^ | - |  |  |
| Alcohol intake | 0.15^†^ | 0.15^†^ | - |  |
| Diet quality | 0.04^†^ | -0.38^†^ | -0.42^†^ | - |

* Pearson correlation coefficients (all such values).

^†^ *P*< 0.001.

**Table S4.** Baseline characteristics of the study population comparing included and excluded participants.

| Characteristics | Included population (n=48,377) | | Excluded population  (n=109,214) | | *P*-value* |
| --- | --- | --- | --- | --- | --- |
|  | n | Values | n | Values |  |
| Age (years) | 48,377 | 50.63 ± 14.56^†^ | 109,214 | 44.20 ± 14.35 | <0.001 |
| Sex (female) | 48,377 | 37,568 (77.66)^‡^ | 109,214 | 84,979 (77.81) | 0.501 |
| Educational level | 48,278 |  | 103,524 |  | <0.001 |
| Less than high school  degree |  | 1,032 (2.14) |  | 4,088 (3.95) |  |
| <2 years after high school  degree |  | 14,108 (31.39) |  | 38,765 (37.45) |  |
| ≥2 years after high school  degree |  | 33,091 (68.61) |  | 60,671 (59.61) |  |
| Smoking status | 48,376 |  | 107,536 |  | <0.001 |
| Never |  | 22,333 (46.17) |  | 48,209 (44.83) |  |
| Former |  | 20,899 (43.20) |  | 36,420 (33.87) |  |
| Current |  | 5,144 (10.63) |  | 22,907 (21.30) |  |
| Physical activity (IPAQ) | 48,314 |  | 93,537 |  | <0.001 |
| Low |  | 11,025 (22.83) |  | 29,916 (31.98) |  |
| Medium |  | 20,248 (41.94) |  | 37,917 (40.54) |  |
| High |  | 17,009 (35.23) |  | 25,704 (27.48) |  |
| Energy intake without alcohol (kcal/day) | 45,080 | 1,787.29 ± 474.28 | 69,150 | 1800.02 ± 493.75 | 0.030 |
| Alcohol intake (g/day) | 45,080 | 7.92 ± 12.01 | 69,150 | 7.34 ± 12.50 | <0.001 |
| Diet quality (sPNNS-GS2; range: −17 to 13.5) | 44,318 | 1.28 ± 3.56 | 59,529 | 1.01 ± 3.55 | <0.001 |
| Body mass index (kg/m^2^) | 48,212 | 23.86 ± 4.34 | 104,512 | 24.43 ± 5.13 | <0.001 |
| Hypertension prevalence and/or medication | 48,377 | 6,400 (13.23) | 107,697 | 10,642 (9.88) | <0.001 |
| Hypercholesterolemia prevalence and/or medication | 48,377 | 8,719 (18.02) | 107,698 | 10,103 (9.38) | <0.001 |
| Hypertriglyceridemia prevalence and/or medication | 48,377 | 1,657 (3.43) | 107,697 | 2,565 (2.38) | <0.001 |
| Family history of diabetes | 47,996 | 9,689 (20.19) | 105,221 | 20,081 (19.08) | <0.001 |
| Depressive symptomatology | 19,464 | 2,359 (12.12) | 16,658 | 2,996 (16.44) | <0.001 |

Abbreviations: IPAQ, International Physical Activity Questionnaire; sPNNS-GS2, simplified Programme National Nutrition Santé - Guidelines Score 2.

^*^*P*-value showing comparisons between included and excluded participants based on chi-square for categorical variables and t-test for quantitative variables.

^†^ mean ± SD (all such values).

^‡^ n (%) (all such values).

**Table S5.** Associations between categories of trait impulsivity and the risk of developing type 2 diabetes.

| Trait impulsivity categories |  | Low | |  | Medium | |  | High | |
| --- | --- | --- | --- | --- | --- | --- | --- | --- | --- |
| Type 2 diabetes |  | HR (95CI%) | *P*-value |  | HR (95CI%) | *P*-value |  | HR (95CI%) | *P*-value |
| Parsimonious model |  | Ref. | **-** |  | 1.16  (0.92, 1.46) | 0.20 |  | 1.41  (0.97, 2.05) | 0.072 |
| Main model |  | Ref. | **-** |  | 1.16  (0.92, 1.46) | 0.20 |  | 1.40  (0.97, 2.04) | 0.074 |

Abbreviations: HR (95%CI), hazard ratio and 95% confidence interval. Cox regression analyses were performed using hazard ratios and 95% CI to assess associations between categories of total trait impulsivity (low category as reference) and the risk of type 2 diabetes incidence over a median follow-up of 8 years in the NutriNet-Santé cohort. Total population (n= 48,377) and type 2 diabetes incident cases (n= 556). Person-years= 297,027 and incidence rate= 1.87 (95% CI: 1.72, 2.03) per 1,000 person-years. Parsimonious model: adjusted for baseline sex and age (time scale). Main model: Parsimonious model + baseline education level (less than high school degree, <2 years after high school degree, ≥2 years after high school degree), smoking status (never, former, current smoker), physical activity (International Physical Activity Questionnaire: high, moderate, low), energy intake without alcohol (kcal/day), alcohol intake (g/day), diet quality (simplified Programme National Nutrition Santé - Guidelines Score 2). Non-proportional hazard risk covariates were corrected by adding a logarithmic time interaction (educational level only).

**Table S6.** Assessment of proportional hazard risk assumptions between total trait impulsivity and risk of developing type 2 diabetes, with and without correction for non-proportional hazard risks covariates.

| Characteristics | *P*-value | |
| --- | --- | --- |
|  | Without correction | With correction |
| Impulsivity | 0.73 | 0.70 |
| Sex (female) | 0.066 | 0.060 |
| Educational level |  |  |
| Less than high school degree | 0.77 | - |
| <2 years after high school degree | Ref. | - |
| ≥2 years after high school degree | <0.001 | - |
| Smoking status |  |  |
| Never | Ref. | Ref. |
| Former | 0.53 | 0.49 |
| Current | 0.43 | 0.39 |
| Physical activity |  |  |
| Low | Ref. | Ref. |
| Medium | 0.71 | 0.69 |
| High | 0.39 | 0.37 |
| Energy intake without alcohol | 0.67 | 0.68 |
| Alcohol intake | 0.99 | 0.97 |
| Diet quality | 0.30 | 0.28 |
| Global test | 0.033 | 0.54 |

The assessment of proportional hazard risk assumptions was tested using the Schoenfeld residuals test in the main model. The correction of non-proportional hazard risk was performed using stratified estimates for significant covariates showing a non-proportional hazard risk assumption at baseline (educational level only).

**Table S7.** Assessment of proportional hazard risk assumptions between impulsivity subfactors and risk of developing type 2 diabetes, with and without correction for proportional hazard risks covariates.

| Impulsivity subfactors | Attention | | Motor | | Non-planning | |
| --- | --- | --- | --- | --- | --- | --- |
|  | *P*-value | | *P*-value | | *P*-value | |
| Characteristic | Without correction | With correction | Without correction | With correction | Without correction | With correction |
| Impulsivity | 0.85 | 0.79 | 0.31 | 0.29 | 0.97 | 0.91 |
| Sex (female) | 0.077 | 0.071 | 0.058 | 0.052 | 0.077 | 0.069 |
| Educational level |  |  |  |  |  |  |
| Less than high school degree | 0.74 | - | 0.79 | - | 0.76 | - |
| <2 years after high school degree | Ref. | - | Ref. | - | Ref. | - |
| ≥2 years after high school degree | <0.001 | - | <0.001 | - | <0.001 | - |
| Smoking status |  |  |  |  |  |  |
| Never | Ref. | Ref. | Ref. | Ref. | Ref | Ref |
| Former | 0.51 | 0.45 | 0.58 | 0.53 | 0.51 | 0.47 |
| Current | 0.41 | 0.37 | 0.47 | 0.43 | 0.41 | 0.38 |
| Physical activity |  |  |  |  |  |  |
| Low | Ref. | Ref. | Ref. | Ref. | Ref | Ref. |
| Medium | 0.70 | 0.69 | 0.70 | 0.68 | 0.71 | 0.70 |
| High | 0.40 | 0.39 | 0.38 | 0.37 | 0.39 | 0.37 |
| Energy intake without alcohol | 0.68 | 0.69 | 0.68 | 0.69 | 0.67 | 0.68 |
| Alcohol intake | 0.99 | 0.97 | 0.99 | 0.96 | 0.99 | 0.96 |
| Diet quality | 0.31 | 0.23 | 0.31 | 0.28 | 0.31 | 0.30 |
| Global test | 0.033 | 0.57 | 0.025 | 0.44 | 0.036 | 0.57 |

The assessment of the proportional hazard risk was tested using the Schoenfeld residuals test for the main model. The correction of non-proportional hazard risk was performed using stratified estimates for significant covariates showing a non-proportional hazard risk assumption at baseline (educational level only).

**Table** **S8.** Associations between total and subfactors of trait impulsivity and risk of developing type 2 diabetes over 8 years using stratified estimates for non-proportional hazard risk covariates.

| Type 2 diabetes | HR (95% CI) | *P*-value |
| --- | --- | --- |
| Total impulsivity | Per 1SD |  |
| Parsimonious model | 1.15 (1.06, 1.25) | 0.001 |
| Main model | 1.10 (1.01, 1.19) | 0.026 |
| Attention | Per 1SD |  |
| Parsimonious model | 1.10 (1.01, 1.20) | 0.025 |
| Main model | 1.07 (0.98, 1.16) | 0.12 |
| Motor | Per 1SD |  |
| Parsimonious model | 1.16 (1.07, 1.26) | <0.001 |
| Main model | 1.13 (1.04, 1.23) | 0.004 |
| Non-planning | Per 1SD |  |
| Parsimonious model | 1.10 (1.02, 1.20) | 0.020 |
| Main model | 1.05 (0.96, 1.14) | 0.30 |

Abbreviations: HR (95% CI), hazard ratio and 95% confidence interval; Per 1SD, per 1 standard deviation increment. Cox regression analyses were performed using hazard ratios and 95% CI to assess associations between 1SD increment of total and subfactors of trait impulsivity and the risk of type 2 diabetes incidence over a median follow-up of 8 years in the NutriNet-Santé cohort. The motor impulsivity subfactor was log10 transformed due to potential non-linear association (*P*= 0.026). Total population (n= 48,377) and type 2 diabetes incident cases (n= 556). Person-years= 297,027 and incidence rate= 1.87 (95% CI: 1.72, 2.03) per 1,000 person-years. Parsimonious model: adjusted for baseline sex and age (time-scale). Main model: Parsimonious model + baseline educational level (less than high school degree, <2 years after high school degree, ≥2 years after high school degree), smoking status (never, former, current smoker), physical activity (International Physical Activity Questionnaire: high, moderate, low), energy intake without alcohol (kcal/day), alcohol intake (g/day), and diet quality (simplified Programme National Nutrition Santé - Guidelines Score 2). Non-proportional hazard risk covariates were corrected using stratified estimates (educational level only).

**Table S9.** Associations between total and subfactors of trait impulsivity and risk of developing type 2 diabetes over 8 years without proportional hazard risk correction.

| Type 2 diabetes | HR (95% CI) | *P*-value |
| --- | --- | --- |
| Total impulsivity | Per 1SD |  |
| Parsimonious model | 1.15 (1.06, 1.25) | 0.001 |
| Main model | 1.10 (1.01, 1.20) | 0.022 |
| Attention | Per 1SD |  |
| Parsimonious model | 1.10 (1.01, 1.20) | 0.025 |
| Main model | 1.07 (0.98, 1.16) | 0.12 |
| Motor | Per 1SD |  |
| Parsimonious model | 1.17 (1.07, 1.27) | <0.001 |
| Main model | 1.13 (1.04, 1.23) | 0.003 |
| Non-planning | Per 1SD |  |
| Parsimonious model | 1.10 (1.02, 1.20) | 0.020 |
| Main model | 1.05 (0.97, 1.14) | 0.25 |

Abbreviations: HR (95% CI), hazard ratio and 95% confidence interval; Per 1SD, per 1 standard deviation increment. Cox regression analyses were performed using hazard ratios and 95% CI to assess associations between 1SD increment of total and subfactors of trait impulsivity and the risk of type 2 diabetes incidence over a median follow-up of 8 years in the NutriNet-Santé cohort. The motor impulsivity subfactor was log10 transformed due to potential non-linear association (*P*= 0.026). Total population (n= 48,377) and type 2 diabetes incident cases (n= 556). Person-years= 297,027 and incidence rate= 1.87 (95% CI: 1.72, 2.03) per 1,000 person-years. Parsimonious model: adjusted for baseline sex and age (time-scale). Main model: Parsimonious model + baseline educational level (less than high school degree, <2 years after high school degree, ≥2 years after high school degree), smoking status (never, former, current smoker), physical activity (International Physical Activity Questionnaire: high, moderate, low), energy intake without alcohol (kcal/day), alcohol intake (g/day), and diet quality (simplified Programme National Nutrition Santé - Guidelines Score 2).

**Table S10.** All models of associations between total and subfactors of trait impulsivity and risk of developing type 2 diabetes over 8 years.

| Type 2 diabetes | HR (95% CI) | *P*-value |
| --- | --- | --- |
| Total impulsivity | Per 1SD |  |
| Model 2 | 1.14 (1.04, 1.23) | 0.003 |
| Model 3 | 1.11 (1.02, 1.21) | 0.012 |
| Model 5 | 1.08 (1.00, 1.18) | 0.055 |
| Model 6 | 1.08 (0.99, 1.17) | 0.071 |
| Model 7 | 1.08 (0.99, 1.18) | 0.075 |
| Model 8 | 1.03 (0.95, 1.12) | 0.52 |
| Attention | Per 1SD |  |
| Model 2 | 1.09 (1.00, 1.18) | 0.044 |
| Model 3 | 1.08 (0.99, 1.17) | 0.083 |
| Model 5 | 1.04 (0.96, 1.13) | 0.33 |
| Model 6 | 1.04 (0.96, 1.13) | 0.34 |
| Model 7 | 1.04 (0.96, 1.13) | 0.35 |
| Model 8 | 1.03 (0.94, 1.12) | 0.53 |
| Motor | Per 1SD |  |
| Model 2 | 1.16 (1.07, 1.26) | 0.001 |
| Model 3 | 1.14 (1.04, 1.24) | 0.003 |
| Model 5 | 1.12 (1.02, 1.21) | 0.009 |
| Model 6 | 1.11 (1.02, 1.20) | 0.016 |
| Model 7 | 1.11 (1.02, 1.21) | 0.017 |
| Model 8 | 1.04 (0.96, 1.13) | 0.37 |
| Non-planning | Per 1SD |  |
| Model 2 | 1.08 (0.99, 1.17) | 0.073 |
| Model 3 | 1.06 (0.97, 1.15) | 0.17 |
| Model 5 | 1.05 (0.96, 1.14) | 0.27 |
| Model 6 | 1.05 (0.96, 1.14) | 0.29 |
| Model 7 | 1.05 (0.96, 1.14) | 0.30 |
| Model 8 | 1.01 (0.93, 1.10) | 0.84 |

Abbreviations: HR (95% CI), hazard ratio and 95% confidence interval; Per 1SD, per 1 standard deviation increment. Cox regression analyses were performed using hazard ratios and 95% CI to assess associations between 1SD increment of total and subfactors of trait impulsivity and the risk of type 2 diabetes incidence over a median follow-up of 8 years in the NutriNet-Santé cohort. The motor impulsivity subfactor was log10 transformed due to potential non-linear association (*P*= 0.026). Total population (n= 48,377) and type 2 diabetes incident cases (n= 556). Person-years= 297,027 and incidence rate= 1.87 (95% CI: 1.72, 2.03) per 1,000 person-years. Parsimonious model (data shown in Fig. 2 & 3): adjusted for baseline sex and age (time-scale). Model 2 (sociodemographics): Parsimonious model + baseline educational level (less than high school degree, <2 years after high school degree, ≥2 years after high school degree). Model 3 (lifestyle): Model 2 + baseline smoking status (never, former, current smoker), physical activity (International Physical Activity Questionnaire: high, moderate, low), energy intake without alcohol (kcal/day), and alcohol intake (g/day). Main model (data shown in Fig. 2 & 3): Model 3 + baseline diet quality (simplified Programme National Nutrition Santé - Guidelines Score 2). Model 5 (personal history of disease): Main model + baseline hypertension prevalence or medication (no, yes), hypercholesterolemia prevalence or medication (no, yes), and hypertriglyceridemia prevalence or medication (no, yes). Model 6 (familiar history of disease): Model 5 + baseline family history of type 2 diabetes (no, yes). Model 7 (depressive symptomatology): Model 6 + baseline depressive symptomatology (Center for Epidemiologic Studies Depression Scale score: no, yes). Model 8 (anthropometrics): Model 7 + baseline body mass index (kg/m^2^). Non-proportional hazard risk covariates were corrected by adding a logarithmic time interaction (educational level only).

**Table S11.** Associations between total and subfactors of trait impulsivity and risk of developing type 2 diabetes over 8 years excluding incident cases in the first 2 years of follow-up.

| Type 2 diabetes | HR (95% CI) | *P*-value |
| --- | --- | --- |
| Total impulsivity | 1SD increment |  |
| Parsimonious model | 1.15 (1.03, 1.28) | 0.010 |
| Main model | 1.09 (0.98, 1.21) | 0.11 |
| Attention | 1SD increment |  |
| Parsimonious model | 1.04 (0.93, 1.16) | 0.49 |
| Main model | 1.01 (0.90, 1.12) | 0.92 |
| Motor | 1SD increment |  |
| Parsimonious model | 1.21 (1.08, 1.34) | 0.001 |
| Main model | 1.17 (1.05, 1.30) | 0.004 |
| Non-planning | 1SD increment |  |
| Parsimonious model | 1.11 (1.00, 1.24) | 0.046 |
| Main model | 1.05 (0.95, 1.17) | 0.33 |

Abbreviations: HR (95% CI), hazard ratio and 95% confidence interval; Per 1SD, per 1 standard deviation increment. Cox regression analyses were performed using hazard ratios and 95% CI to assess associations between 1SD increment of total and subfactors of trait impulsivity and the risk of type 2 diabetes incidence over a median follow-up of 8.17 (interquartile range: 5.82-8.54) years in the NutriNet-Santé cohort. The motor impulsivity subfactor was log10 transformed due to potential non-linear association (*P*= 0.027). Total population (n= 41,389) and type 2 diabetes incident cases (n= 342). Person-years= 290,692 and incidence rate= 1.18 (95% CI: 1.06, 1.31) per 1,000 person-years. Parsimonious model: adjusted for baseline sex and age (time-scale). Main model: adjusted for Parsimonious model further baseline educational level (less than high school degree, <2 years after high school degree, ≥2 years after high school degree), smoking status (never, former, current smoker), physical activity (International Physical Activity Questionnaire: high, moderate, low), energy intake without alcohol (kcal/day), alcohol intake (g/day), and diet quality (simplified Programme National Nutrition Santé - Guidelines Score 2). Non-proportional hazard risk covariates were corrected by adding a logarithmic time interaction (educational level only).

**REFERENCES**

1. Steinberg L, Albert D, Cauffman E, Banich M, Graham S, Woolard J. Age differences in sensation seeking and impulsivity as indexed by behavior and self-report: Evidence for a dual systems model. Dev Psychol. 2008;44(6):1764–78.

2. Moustafa AA, Tindle R, Frydecka D, Misiak B. Impulsivity and its relationship with anxiety, depression and stress. Compr Psychiatry. 2017;74(1):173–9.

3. Haffner SM. Epidemiology of Type 2 Diabetes: Risk Factors. Diabetes Care. 1998;21(Supplement_3):C3–6.

4. Cross CP, Copping LT, Campbell A. Sex differences in impulsivity: A meta-analysis. Psychol Bull. 2011;137(1):97–130.

5. Kautzky-Willer A, Leutner M, Harreiter J. Sex differences in type 2 diabetes. Diabetologia. 2023;66(6):986–1002.

6. Schwartz JA, Connolly EJ, Alsolami A. Within-Individual Changes in Impulsivity and Sensation Seeking from Childhood to Early Adulthood and Educational Attainment. J Youth Adolesc. 2022;51(11):2190–204.

7. Bellou V, Belbasis L, Tzoulaki I, Evangelou E. Risk factors for type 2 diabetes mellitus: An exposure-wide umbrella review of meta-analyses. PLoS ONE. 2018;13(3):e0194127.

8. Kale D, Stautz K, Cooper A. Impulsivity related personality traits and cigarette smoking in adults: A meta-analysis using the UPPS-P model of impulsivity and reward sensitivity. Drug Alcohol Depend. 2018;185(1):149–67.

9. Bos J, Hayden MJ, Lum JAG, Staiger PK. UPPS-P impulsive personality traits and adolescent cigarette smoking: A meta-analysis. Drug Alcohol Depend. 2019;197(1):335–43.

10. Dendup T, Feng X, Clingan S, Astell-Burt T. Environmental Risk Factors for Developing Type 2 Diabetes Mellitus: A Systematic Review. Int J Environ Res Public Health. 2018;15(1):78.

11. Castañer M, Aiello S, Prat Q, Andueza J, Crescimanno G, Camerino O. Impulsivity and physical activity: A T-Pattern detection of motor behavior profiles. Physiol Behav. 2020;219:112849.

12. Booth FW, Roberts CK, Laye MJ. Lack of exercise is a major cause of chronic diseases. Compr Physiol. 2012;2(2):1143–211.

13. Mõttus R, McNeill G, Jia X, Craig LCA, Starr JM, Deary IJ. The associations between personality, diet and body mass index in older people. Health Psychol. 2013;32(4):353–60.

14. Bénard M, Bellisle F, Kesse-Guyot E, Julia C, Andreeva VA, Etilé F, et al. Impulsivity is associated with food intake, snacking, and eating disorders in a general population. Am J Clin Nutr. 2019;109(1):117–26.

15. Villegas R, Shu XO, Yang G, Matthews CE, Li H, Cai H, et al. Energy balance and type 2 diabetes: A report from the Shanghai Women’s Health Study. Nutr Metab Cardiovasc Dis. 2009;19(3):190–7.

16. van de Wiel A. Diabetes mellitus and alcohol. Diabetes Metab Res Rev. 2004;20(4):263–7.

17. Gómez-Martínez C, Babio N, Júlvez J, Nishi SK, Fernández-Aranda F, Martínez-González MÁ, et al. Impulsivity is longitudinally associated with healthy and unhealthy dietary patterns in individuals with overweight or obesity and metabolic syndrome within the framework of the PREDIMED-Plus trial. Int J Behav Nutr Phys Act. 2022;19(1):101.

18. Chaltiel D, Adjibade M, Deschamps V, Touvier M, Hercberg S, Julia C, et al. Programme National Nutrition Santé – guidelines score 2 (PNNS-GS2): development and validation of a diet quality score reflecting the 2017 French dietary guidelines. Br J Nutr. 2019;122(2):331–42.

19. Emery RL, Levine MD. Questionnaire and behavioral task measures of impulsivity are differentially associated with body mass index: A comprehensive meta-analysis. Psychol Bull. 2017;143(8):868–902.

20. Fuemmeler BF, Østbye T, Yang C, McClernon FJ, Kollins SH. Association between attention-deficit/hyperactivity disorder symptoms and obesity and hypertension in early adulthood: a population-based study. Int J Obes (Lond). 2011;35(6):852–62.

21. Sutin AR, Terracciano A, Deiana B, Uda M, Schlessinger D, Lakatta EG, et al. Cholesterol, triglycerides, and the Five-Factor Model of personality. Biol Psychol. 2010;84(2):186–91.

22. Eriksson A-K, Gustavsson JP, Hilding A, Granath F, Ekbom A, Östenson C-G. Personality traits and abnormal glucose regulation in middle-aged Swedish men and women. Diabetes Res Clin Pract. 2012;95(1):145–52.

23. Scott RA, Langenberg C, Sharp SJ, Franks PW, Rolandsson O, Drogan D, et al. The link between family history and risk of type 2 diabetes is not explained by anthropometric, lifestyle or genetic risk factors: The EPIC-InterAct study. Diabetologia. 2013;56(1):60–9.

24. Fields SA, Schueler J, Arthur KM, Harris B. The Role of Impulsivity in Major Depression: A Systematic Review. Curr Behav Neurosci Rep. 2021;8(1):38–50.

25. Graham EA, Deschênes SS, Khalil MN, Danna S, Filion KB, Schmitz N. Measures of depression and risk of type 2 diabetes: A systematic review and meta-analysis. J Affect Disord. 2020;265(1):224–32.

26. Fuhrer R., Rouillon F. La version française de l’échelle CES-D (Center for Epidemiologic Studies-Depression Scale). Description et traduction de l’échelle d’autoévaluation. Psychiatry and Psychobiology. 1989;4(3):163–6.
