# Supplementary material for: Trait impulsivity is associated with an increased risk of type 2 diabetes incidence in adults over 8 years of follow-up: results from the NutriNet-Santé cohort
Source: BMC Med. 2024 Aug 15;22:332. doi: 10.1186/s12916-024-03540-7 (PMC11328429; doi:10.1186/s12916-024-03540-7)
Supplement: Supplementary file 3 — Additional file 3: Fig. S1 Cumulative hazards between categories of total trait impulsivity and risk of developing type 2 diabetes. Fig. S2 Restricted cubic splines between trait impulsivity and risk of developing type 2 diabetes. Fig. S3 Mediation analysis of body mass indexlevels in the associations between total trait impulsivity and the risk of developing type 2 diabetes [file 12916_2024_3540_MOESM3_ESM.docx]

**Figure S1.** Cumulative hazards between categories of total trait impulsivity and risk of developing type 2 diabetes.

Cox regression analyses were performed using hazard ratios and 95% CI to assess associations between 1SD increment of trait impulsivity and the risk of type 2 diabetes incidence over a median follow-up of 8 years in the NutriNet-Santé cohort. Cumulative hazards were estimated for low (<52), medium (≥52 and ≤71) and high impulsivity (>71) categories using the Barratt Impulsiveness Scale 11 questionnaire. Analyses are performed on the main model that is adjusted at baseline for sex and age (time-scale), educational level (less than high school degree, <2 years after high school degree, ≥2 years after high school degree), smoking status (never, former, current smoker), physical activity (International Physical Activity Questionnaire: high, moderate, low), energy intake without alcohol (kcal/day), alcohol intake (g/day), and diet quality (simplified Programme National Nutrition Santé - Guidelines Score 2).

**Figure S2.** Restricted cubic splines between trait impulsivity and risk of developing type 2 diabetes.

**
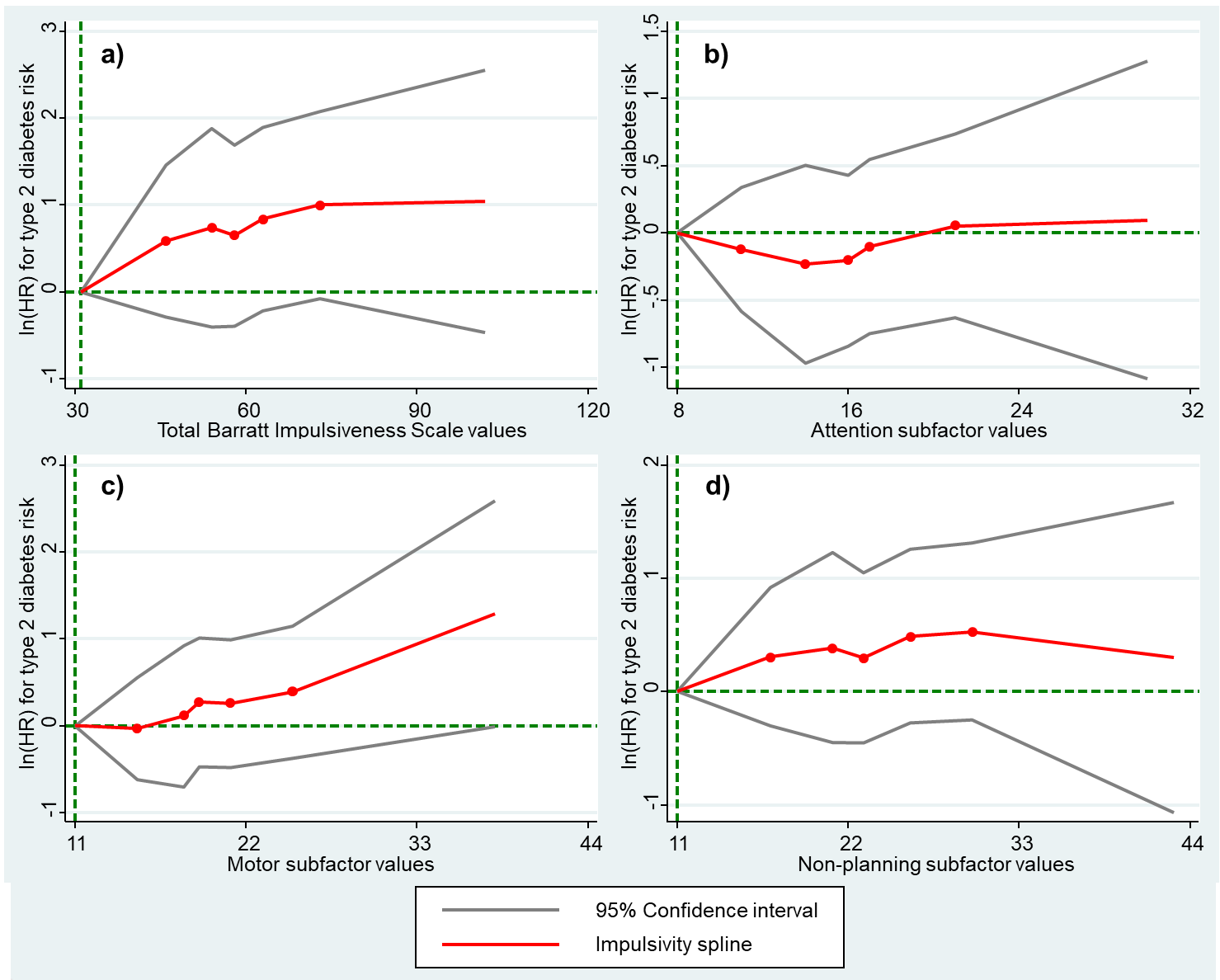
**

**a)** Total trait impulsivity. **b)** Attention impulsivity subfactor. **c)** Motor impulsivity subfactor. **d)** Non-planning impulsivity subfactor. Cox regression analyses were performed using hazard ratios and 95% CI to assess associations between trait impulsivity values and the risk of type 2 diabetes incidence over a median follow-up of 8 years in the NutriNet-Santé cohort. Spline plot modeling the association between total trait impulsivity and type 2 diabetes risk was obtained using restricted cubic spline and piecewise cubic polynomials across 5 adjacent knots of trait impulsivity. Knots are represented with a red dot for total impulsivity: 46, 54, 58, 63, 73; for attentional subfactor: 11, 14, 16, 17, 21; for motor subfactor: 15, 18, 19, 21, 25; for non-planning subfactor: 17, 21, 23, 26, 30. Main model adjusted at baseline for sex and age (time-scale), educational level (less than high school degree, <2 years after high school degree, ≥2 years after high school degree), smoking status (never, former, current smoker), physical activity (International Physical Activity Questionnaire: high, moderate, low), energy intake without alcohol (kcal/day), alcohol intake (g/day), and diet quality (simplified Programme National Nutrition Santé - Guidelines Score 2). Non-proportional hazard risk covariates were corrected by adding a logarithmic time interaction (educational level only). *P*-value for the non-potential linear association for total impulsivity= 0.11, attention= 0.32, motor= 0.026, and non-planning= 0.51.

**Figure S3.** Mediation analysis of body mass index (BMI) levels in the associations between total trait impulsivity and the risk of developing type 2 diabetes.

**
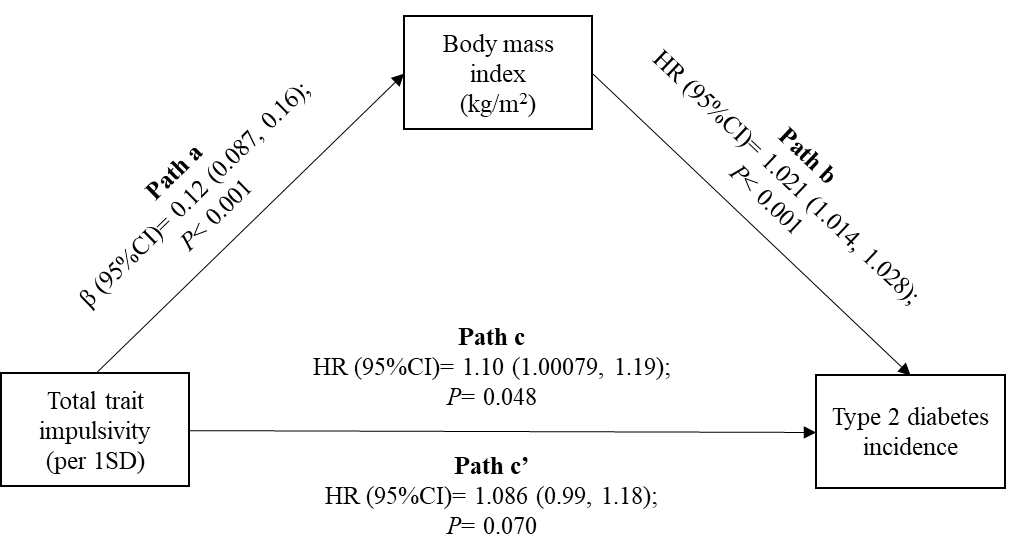
**

**Path a.** Linear association between impulsivity and BMI. **Path b.** Pure indirect effect. **Path c’.** Controlled direct effect fixing BMI values to its mean (23.86 kg/m^2^). **Path c.** Total effect. Abbreviations: β (95% CI), beta coefficients and 95% confidence intervals; HR (95% CI), hazard ratio and 95% confidence interval; per 1SD, per 1 standard deviation increment. Mediation analyses were performed establishing a linear association between total trait impulsivity (exposure) and BMI (mediator), and Cox regression models on type 2 diabetes incidence (outcome). Total population (n= 48,377) and type 2 diabetes incident cases (n= 556). Person-years= 297,027 and incidence rate= 1.87 (95% CI: 1.72, 2.03) per 1,000 person-years. Models were adjusted for baseline sex, age (time scale), education level (less than high school degree, <2 years after high school degree, ≥2 years after high school degree), smoking status (never, former, current smoker), physical activity (International Physical Activity Questionnaire: high, moderate, low), energy intake without alcohol (kcal/day), alcohol intake (g/day), and diet quality (simplified Programme National Nutrition Santé - Guidelines Score 2).
